# Comprehensive assessment of coronary pulse wave velocity in anesthetized pigs

**DOI:** 10.14814/phy2.14424

**Published:** 2020-05-12

**Authors:** Andrei Cividjian, Brahim Harbaoui, Carole Chambonnet, Jeanne‐Marie Bonnet, Christian Paquet, Pierre‐Yves Courand, Pierre Lantelme

**Affiliations:** ^1^ Hospices Civils de Lyon Fédération de Cardiologie Croix‐Rousse ‐ Lyon‐Sud Lyon France; ^2^ Univ Lyon INSA‐Lyon Université Claude Bernard Lyon 1 UJM‐Saint Etienne CNRS Inserm CREATIS UMR 5220, U1206 Lyon France; ^3^ Alpha‐2 Ltd Lyon France; ^4^ i‐COR Technologies Lyon France; ^5^ Univ Lyon VetAgro Sup APCSe Marcy l’Etoile France

**Keywords:** coronary physiology, coronary stiffness, pulse wave velocity

## Abstract

**Background:**

Coronary stiffness represents a new paradigm for interventional cardiology and can be assessed by coronary pulse wave velocity (CoPWV). Assessing CoPWV is complex because of the coexistence of backward and forward waves.

**Objectives:**

Evaluate the feasibility, repeatability, and capacity of methods assessing CoPWV to detect predictable velocity changes.

**Methods:**

CoPWV was measured from distal and proximal pressure guidewires in the left anterior descending artery of 10 pigs under general anesthesia. Four methods were studied: the tangent intersection method applied to the forward (FW) and backward (BK) waves, as well as the dicrotic notch (DIC) and template matching (TM) methods. All were evaluated at baseline, during various arterial pressure and heart rate conditions, during simulated flow limitation (balloon inflation), and after increasing coronary stiffness (stent insertion).

**Results:**

All the methods were significantly different between them (*p* ≤ .05) showing a systematic trend toward higher CoPWV when compared to the FW method (.05 < *p*<.10). Results were found to be significantly correlated only between the BK and FW methods and between the DIC and TM methods (*p* ≤ .05). CoPWV increased with arterial pressure increase, this increase being significant for the DIC and TM methods and partly for the FW method (*p* ≤ .05). Conversely, heart rate had no systematic impact on CoPWV. The lowest variability was found for the DIC and TM methods (*p* ≤ .05). Only the BK and TM methods remained applicable during flow limitation; stent increased CoPWV when measured by the BK method only (*p* ≤ .05).

**Conclusion:**

Although CoPWV can be measured by various methods, the BK and TM methods seem the most appropriate for clinical studies.

## Graphical abstract

Four methods for measuring coronary pulse wave velocity (CoPWV) were compared in animal (*n* = 10) using two regular pressure guidewires. During decompression phase a trend exists for higher CoPWV and repeatability is better than during compression phase. When coronary blood flow is reduced, known methods assessing CoPWV are not applicable and only the template matching and a new “backward wave” method yields reliable results.

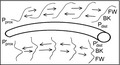

## 1 INTRODUCTION

Implementation of coronary physiology, that is, fractional flow reserve (FFR) or, more recently, instantaneous wave‐free ratio (iFR), has been a major breakthrough in the cath lab (Davies et al., 2017; De Bruyne et al., 2012). In cases of intermediate coronary stenosis, the FFR and iFR indices are used as surrogates for coronary flow reserve (CFR), helping physicians during the decision‐making process. Although an FFR > 0.8 identifies a group of patients with a low absolute risk of major adverse cardiac event (MACE) (Barbato et al., 2016), the value of a decision based on this cut‐off alone is far from optimal as FFR and CFR, which both aim at identifying ischemia‐prone lesions, are in disagreement in almost 30% of cases (Garcia et al., 2019; van de Hoef et al., 2014). Furthermore, these indices are not suitable for predicting acute plaque complication. In this context, new coronary physiology indices are required to improve the diagnosis of stable and unstable coronary artery disease. Coronary stiffness could represent such an index.

The matrix of the plaque and the structures surrounding the lumen of the vessels represent major determinants of mechanical properties of arteries and can explain how a stenosis behaves under increased pressure (such as during exercise stress) or resists to cyclic stretch. This may be important to determine how the stenosis impacts the flow increase. For instance, a “soft” stenosis behaves like a collapsible tube and the lumen diameter increases with flow increase (Conrad, 1969). Coronary stiffness may also impact the assessment of stenosis by pressure indices: FFR is erroneously higher when a fully distensible model of artery is used, that is, when stiffness is lower (Yong et al., 2017). Above all, coronary stiffness may influence the risk of acute coronary events: stiffer (Harbaoui, Courand, Cividjian, & Lantelme, 2017) or calcified (Criqui et al., 2014; Hou et al., 2012) coronary arteries are associated with lower risk of cardiovascular events. It follows that coronary stiffness is an attractive concept in the field of interventional cardiology in addition to pressure and flow indices.

Pulse wave velocity (PWV) in a uniform arterial segment is a surrogate for stiffness (Avolio, 2013; Lieber, 2000) and can be measured at the coronary level (CoPWV) by obtaining two simultaneous pressure measurements at a sufficient distance from one another. However, CoPWV has not yet been developed as a clinical tool, probably because of technical difficulties in signal analysis due to heart being itself a contractile organ. The present study therefore sought to assess, in an animal model, the feasibility of CoPWV measurements using different methods based on pressure wave characterization, but also the robustness of these measurements in well‐controlled experimental conditions.

## 2 METHODS

### 2.1 Methods for measuring CoPWV

**FIGURE 1:**
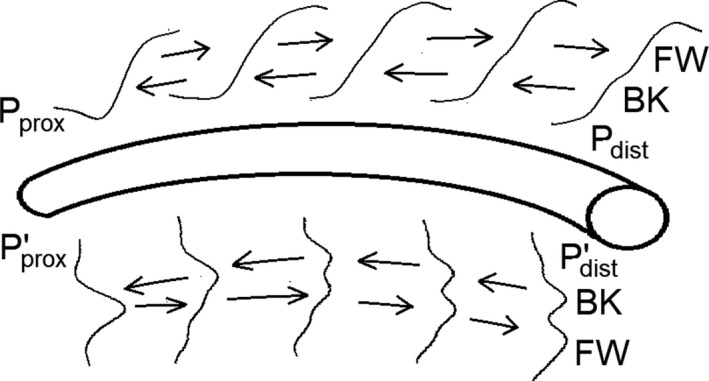
Morphology of the intra‐coronary pressure at the proximal and distal levels in normal conditions. The propagation of the backward (BK) and forward (FW) pressure waves is shown at several positions across the coronary artery between proximal and distal locations on the pressure waves (upward: *P*_prox_ and *P*_dist_) and on their first derivatives (downward: *P*’_prox_ and *P*’_dist_)

**FIGURE 2:**
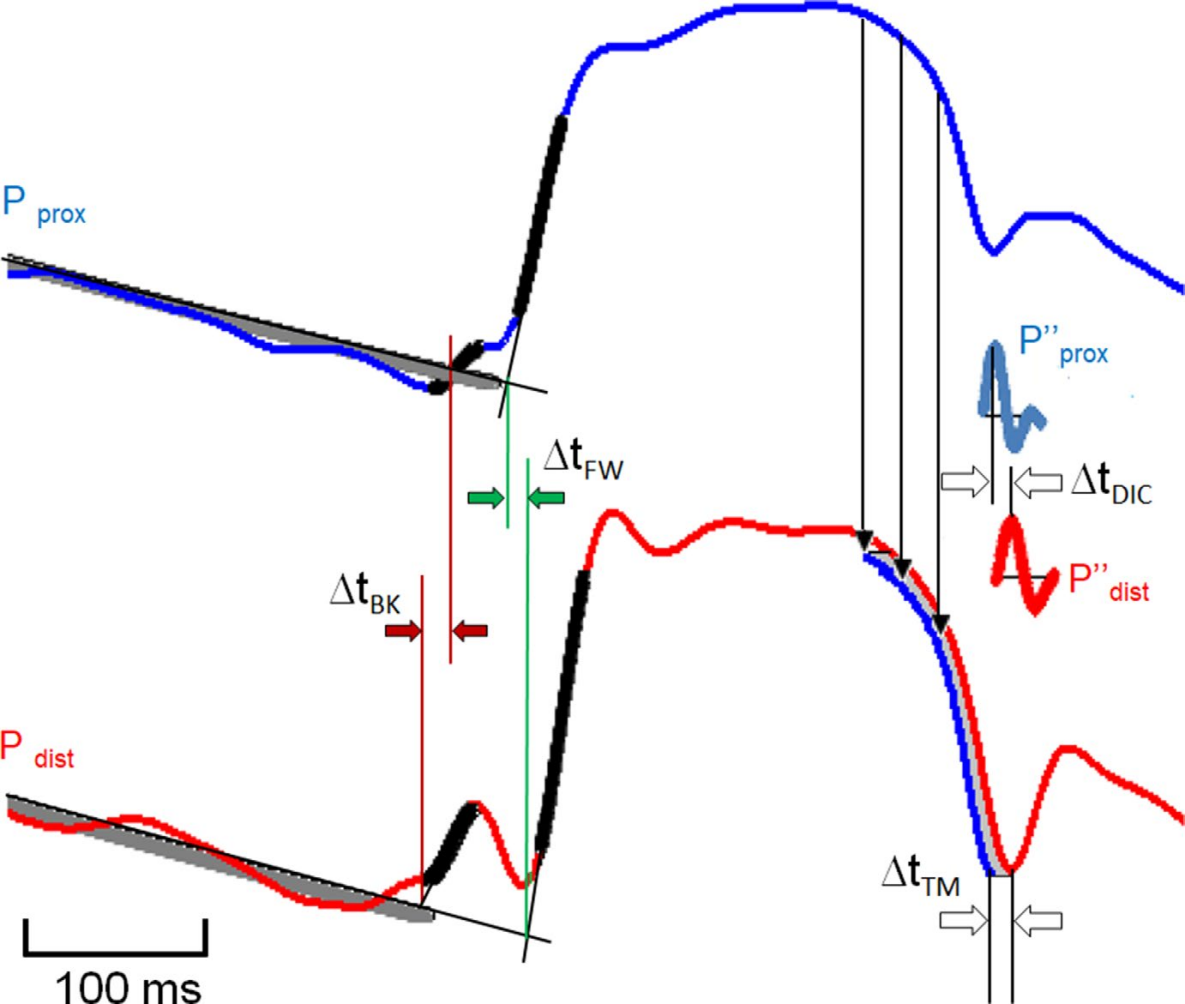
Illustration of the four methods used for measuring CoPWV. The transit time (∆*t*) of the pressure wave between the proximal (*P*_prox_) and the distal (*P*_dist_) sites can be calculated in four different ways; ∆*t*_FW_, tangent intersection method applied to the forward pressure (FW) rise (green lines); ∆*t*_BK_, tangent intersection method applied to the backward (BK) pressure rise (brown arrows); ∆*t*_DIC_, maximum of the second derivative corresponding to the dicrotic notch (DIC) (black lines); ∆*t*_TM_, template matching (TM) between two segments after a rescaling (blue lines). In all cases, CoPWV is calculated from the ratio ∆*t*/*d*; *d* is the distance between proximal and distal recording sites

Measuring CoPWV is challenging because two pressure waves travel in the coronary tree during myocardial contraction, contrary to what is found in other arteries (Figure 1a): one is generated by the ventricular ejection and is responsible for systolic upstroke (hereafter called “*forward pressure wave*”); the other is generated by the compression of microvasculature during isovolumic cardiac contraction and travels in the opposite direction (hereafter called “*backward pressure wave*”) (Davies et al., 2006; Sen, Petraco, Mayet, & Davies, 2014). At the distal side, the two waves are clearly separated and at the proximal side these overlap (Figure 1). The methods used in this study are described in Figure 2 which illustrates characteristic points of the pressure wave during pressure upstroke (compression phase) and pressure fall (decompression phase). The first two methods are obtained during the compression phase; the forward wave (FW) method is the classical foot‐to‐foot method using the tangent intersection to identify the onset of pressure rise (Chiu, Arand, Shroff, Feldman, & Carroll, 1991) applied to the forward wave; the backward wave (BK) method uses the tangent intersection to identify the onset of pressure rise applied to the backward wave. The two other methods are obtained during the decompression phase of the cardiac cycle; the dicrotic notch (DIC) method uses this characteristic point of the pressure wave (Rolandi et al., 2014), and the template matching (TM) method uses the fall in pressure during the decompression phase (Harbaoui et al., 2017).

### 2.2 Experimental set‐up and study design

Experiments were conducted in 10 young (2.5–3.5 months old) female pigs, weighing a median [interquartile range, IQR] of 49 [46.3–50.3] kg, under general anesthesia. The experimental set‐up, which is detailed in the Appendix, was approved by the ethics committee of the Ministry in charge of agriculture (n 2017042115139177), and has therefore been performed in accordance with the ethical standards laid down in the 1964 Declaration of Helsinki and its later amendments. Heart rate (HR) was controlled by pacing through the jugular vein and intravenous esmolol infusion (Baxter Healthcare; up to 260 mg/h). Mean arterial pressure (MAP) changes were obtained using an intravenous infusion of norepinephrine (Mylan; up to 1.8 mg/h) and isosorbide dinitrate (Sanofi‐Aventis; up to 32 mg/h), as needed. Two concomitant intra‐coronary pressure signals were obtained using two identical FFR guidewires (Aeris™, Abbott), each connected to a distinct FFR integrated system (Quantien™). CoPWV was measured in the left anterior descending (LAD) coronary artery in the following conditions:

1. *Spontaneous baseline*: The two guidewires were first superimposed in the proximal LAD coronary artery in order to equalize the signals and avoid deviation. Then, while one guidewire (“proximal”) was kept in the initial proximal position, the other (“distal”) was positioned distally in the LAD coronary artery. Both guidewires were kept in the same position throughout the following steps. The distance between the two external connectors was measured with a millimeter precision ruler and was assumed to be equal to the distance between the proximal and the distal pressure sensors. The mean value and beat‐to‐beat variability (coefficient of variation, CV) of the CoPWV were calculated in baseline conditions.
2. *Hemodynamic conditions*: three levels of HR (90, 110, and 130 bpm) and aortic MAP (60, 80, and 100 mmHg) were imposed in a non‐predefined order, following as closely as possible the current MAP which was more difficult to stabilize in the imposed condition. One condition (110 bpm, 80 mmHg) was repeated at periods separated by more than 20 min for assessment of period‐to‐period variability (CV) and repeatability (intra‐class correlation, ICC).
3. *Induced flow limitation*/*coronary flow limitation*: at fixed HR and aortic MAP (110 bpm, 80 mmHg), an additional regular wire and an intravascular ultrasound (IVUS) probe (Opticross™, Boston Scientific) were inserted to assess the cross‐sectional area of the mid LAD. Then, an angioplasty balloon of appropriate size, inflated at two different pressures, was inserted to resemble as closely as possible a mild (25% luminal surface decrease) and moderate (50% luminal surface decrease) flow limitation. The hemodynamic impact of flow limitation was confirmed by measuring the FFR. Hyperemia was achieved by intracoronary injection of a 150 µg of adenosine as described previously (Toth et al., 2016).
4. *Increased stiffness*: A stent was implanted in the mid LAD between the proximal and distal pressure sensors. The diameter of the stent (Synergy™, Boston Scientific) was chosen according to the mid LAD diameter measured by IVUS.

### 2.3 Data acquisition and analysis

Intra‐coronary proximal (*P*_prox_) and distal (*P*_dist_) pressures from the analog output of the two FFR consoles were sampled at 5 kHz using an analog/digital acquisition board (KUSB 3100, Keithley‐Tektronix) and software (RECAN, Alpha‐2). During acquisition, the acquired waveforms were digitally filtered as described in the Appendix. The acquired signals were pre‐processed off‐line using RECAN as detailed in the Appendix.

CoPWV was measured off‐line using a software library (i‐COR, Pulsalys ‐ Université de Lyon/ Hospices Civils de Lyon and i‐COR Technologies) linked to RECAN. i‐COR library computed, for each cardiac cycle, the pressure‐wave transit delay across the artery between the two pressure sensors using the four concomitant methods detailed above. The transit delays were automatically filtered as described in the Appendix and CoPWV was calculated by dividing the filtered delays by the distance between the pressure sensors. The final CoPWV was the mean of all the filtered CoPWV obtained during a 1‐min period.

### 2.4 Statistical analysis

Data are expressed as median [IQR]. As not all data had normal distribution, non‐parametric methods for paired samples were used for comparisons. A Wilcoxon test was used when two comparisons were performed, and a Friedman test followed by the Wilcoxon test were used when more than two comparisons were performed. For the spontaneous baseline step, linear regressions between CoPWV assessed by all possible methods two‐by‐two were computed. Similarly, Bland‐Altman plots were obtained for all methods two‐by‐two, by plotting differences between CoPWV assessed by two methods against their mean values; the mean and the standard deviation (*SD*) of these differences were used as indices of dispersion. Existance of a proportional bias was tested using linear regression analysis. After verification of the proportionality between *SD* and mean of the CoPWV (significant and positive coefficient of linear regression), variability was assessed for all four methods using the CV of CoPWV within the same 1‐min period (called “beat‐to‐beat”: between 90 and 130 values, depending on the HR) or between two different 1‐min periods (called “period‐to‐period”: 2 values). A logarithmic transformation of the CV was used prior to statistical analysis. Repeatability between two different 1‐min periods was assessed using the ICC. As the onset of the forward wave is the established characteristic point used for the determination of the pressure transit time for the PWV calculation (Chiu et al., 1991), the FW method was considered the reference method for the statistical tests applied to the CoPWV and its variability, excepting the agreements (Bland‐Altman) and linear regressions for whom no assumption of reference method was made. A test with *p* ≤ .05 was considered significant. The data were analyzed using SPSS software (version 21, IBM Corp).

## 3 RESULTS

One pig died at the beginning of the flow limitation step, because of LAD thrombosis resulting in ventricular fibrillation with resuscitation failure. Thus, the first two steps were carried out in all ten pigs, whereas steps 3 and 4 could be carried out in nine pigs only. The results for each experimental condition are presented below.

### 3.1 Condition 1: spontaneous baseline

The median [IQR] baseline aortic MAP was 61.5 [55.8–71.8] mmHg and that of HR was 85 [75.2–98.7] bpm. The four methods computing CoPWV yielded significantly different values (Friedman test: *p* ≤ .05); there was a systematic trend towards higher CoPWV for all methods when compared to the FW method (Wilcoxon test, as compared to the FW method: *p* = .06 for BK, *p* = .07 for DIC, and *p* = .09 for TM). Beat‐to‐beat variability was significantly higher for the BK and DIC methods as compared to the FW method, while period‐to‐period variability was significantly lower for decompression methods (DIC and TM) as compared to the FW method. The highest period‐to‐period repeatability was observed for the TM method (Table 1).

**TABLE 1:** CoPWV values obtained by the four methods

	Compression method		Decompression		
	Forward (FW)	Backward (BK)	Dicrotic Notch (DIC)	Template matching (TM)	Friedman
Median [IQR] CoPWV (m/s)					
Baseline	4.6 [4.1–6.1]	5.3 [3.9–7.0]	5.8 [4.6–6.9]	6.3 [3.9–7.4]	*p* ≤ .05
Variability: median [IQR] CV of CoPWV (%)					
Beat‐to‐beat	9.6 [8.5–13.7]	15.2 [13.9–17.8]*	13.4 [11.1–17.8]*	10.3 [8.3–12.9]	*p* ≤ .05
Period‐to‐period	10.1 [4.3–20.3]	12.8 [7.1–35.3]	4.8 [1.6–14.3]*	3.5 [0.7–13.8]*	*p* ≤ .05
Period ‐to‐period repeatability: Mean [95%CI] ICC					
Single measures	0.72 [0,14–0,94]	0,74 [0.29–0,93]	0.72 [0,24–0.92]	0.79 [0.39–0.94]	
Average measures	0.84 [0.25–0.97]	0.85 [0.44–0.96]	0.84 [0.39–0.96]	0.88 [0.56–0.97]	

Note

Coronary pulse wave velocity (CoPW) obtained in baseline conditions. Beat‐to‐beat variability was obtained in spontaneous baseline conditions. Period‐to‐period variability and repeatability were obtained during two repeated conditions with similar heart rate and mean arterial pressure conditions and expressed as median [IQR]. A logarithmic transformation of the CV was used before the statistical analysis.

Abbreviations: CV, coefficient of variation; ICC, intra‐class correlation.

* *p* ≤ .05 as compared to FW.

**FIGURE 3:**
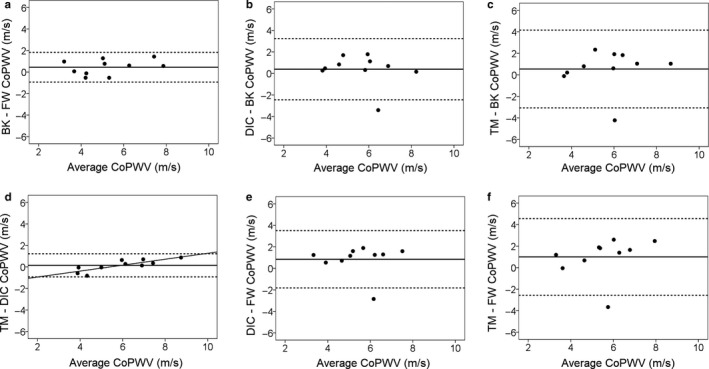
Bland–Altman plots comparing all the possible combinations between the four analysis methods in spontaneous baseline conditions: method BK versus FW (a), method DIC versus BK (b), method TM versus BK (C), method TM versus DIC (d), method DIC versus FW (e) and method TM versus FW (F). Horizontal lines indicate mean and 95% confidence interval (±1.96 *SD*). The smallest confidence interval was observed between the method TM and DIC (d) and between the methods BK and FW (a). The only proportional bias was observed for the plot between the method TM ans DIC (D)

CoPWV estimated with the BK method was significantly correlated with that obtained with the FW method (*r* ^2^ = .81, *p* < .001) but not with the others; CoPWV obtained with the DIC method was significanty correlated with that obtained with the TM method (*r* ^2^ = .96, *p* < .001). The lowest Bland‐Altman dispersions were observed for these comparisons. The only proportional bias was observed between the DIC and the TM methods (Table 2; Figure 3).

**TABLE 2:** Agreement between methods computing CoPWV during spontaneous baseline

Pair of compared methods	Correlation coefficient *r* ^2^	Linear regression *p*	Bland‐Altman dispersion mean (m/s)	Bland‐Altman dispersion *SD* (m/s)	Bland‐Altman proportional bias (Y/N)
BK ‐ FW	**.81**	**<.001**	0.45	0.71	N
BK ‐ DIC	.33	.08	0.40	1.45	N
BK ‐ TM	.22	.18	0.55	1.85	N
FW ‐ DIC	.31	.1	0.85	1.36	N
FW ‐ TM	.18	.23	1.00	1.82	N
DIC ‐ TM	**.96**	**<.001**	0.15	0.55	Y

Note

Abbreviations: BK, backward wave method; DIC, dicrotic notch method; FW, forward wave method; TM, template matching.

Lines in bold corespond to the combinations with the highest correlation coefficient and the lowest dispersion SD

### 3.2 Condition 2: variable hemodynamic conditions

The BK method did not detect the backward wave in the low blood pressure condition (aortic MAP 60 mmHg) and therefore CoPWV was not calculated. There was no systematic significant effect of HR on the CoPWV for each pressure level and each evaluated method; a significant effect was observed with the TM method for aortic MAP = 80 mmHg, and a trend was observed with the BK (*p* = .09) and DIC (*p* = .08) methods for aortic MAP = 80 mmHg. Individual HR values were therefore pooled for each pressure level and each evaluated method. Pooled CoPWV increased for all four methods with increasing aortic MAP, this increase reaching statistical significance for the methods FW, DIC, and TM when aortic MAP increased from 60 to 80 mmHg and for the methods DIC and TM when aortic MAP increased from 80 to 100 mmHg. Of note, the intra‐coronary MAP was slightly lower than the aortic MAP (Table 3).

**TABLE 3:** Impact of heart rate and mean arterial pressure on CoPWV assessed by the four methods

Methods	Median [IQR] CoPWV			
	HR (bpm)	MAP_a_ 60 mmHg	MAP_a_ 80 mmHg	MAP_a_ 100 mmHg
		MAP_c_ 51.9 mmHg	MAP_c_ 66.4 mmHg	MAP_c_ 83.7 mmHg
Forward (FW)	90	4.2 [3.6–5.0]	7.1 [5.0–8.6]	5.3 [3.5–9.5]
	110	4.3 [3.8–5.5]	5.8 [4.0–11.0]	7.3 [5.8–8.1]
	130	4.0 [3.6–4.6]	5.9 [3.5–7.0]	8.3 [5.9–13.0]
	**Pooled**	**4.1 [3.8–4.9]**	**6.0 [4.2–7.7]** *	**7.3 [5.3–9.2]**
Backward (BK)	90	–	4.6 [3.7–7.6]	4.2 [3.8–11.2]
	110	–	4.6 [3.7–7.5]	7.4 [3.4–12.5]
	130	–	7.9 [5.7–9.5]	8.1 [4.5–12.4]
	**Pooled**	–	**5.1 [4.1–9.0]**	**6.1 [3.9–12.0]**
Dicrotic Notch (DIC)	90	5.3 [4.5–6.3]	6.6 [5.6–8.6]	8.5 [7.5–9.3]
	110	5.2 [4.6–6.4]	7.9 [6.2–8.8]	8.7 [7.1–9.3]
	130	5.7 [4.5–6.3]	7.8 [5.8–8.5]	8.6 [7.9–9.5]
	**Pooled**	**5.4 [4.6–6.3]**	**7.7 [5.9–8.5]** *	**8.6 [7.8–9.3]**
Template Matching (TM)	90	5.4 [4.3–6.4]	8.0 [6.2–8.9]	9.2 [7.2–10.3]
	110	5.2 [4.3–6.8]	8.1 [6.7–9.6]	8.9 [6.8–10.4]
	130	5.9 [4.3–6.7]	7.9 [6.0–9.1]	9.3 [7.2–10.1]
	**Pooled**	**5.5 [4.4–6.7]**	**7.9 [6.2–9.0]** *	**9.0 [7.2–10.2]** ^†^

Note

Data are presented as median [interquartile range]. CoPWV could not be calculated by BK method at 60 mmHg.

Abbreviations: bpm, beats per minute; CoPWV, coronary pulse wave velocity; HR, heart rate; MAP_a_, aortic mean arterial pressure; MAP_c_, coronary mean arterial pressure. Lines in bold correspond to pooled CoPWV at a given MAP_a_.

* *p* ≤ .05 versus MAP_a_ = 60 mmHg.

^†^ *p* ≤ .05 versus MAP_a_ = 80 mmHg.

### 3.3 Condition 3: flow Limitation

**FIGURE 4:**
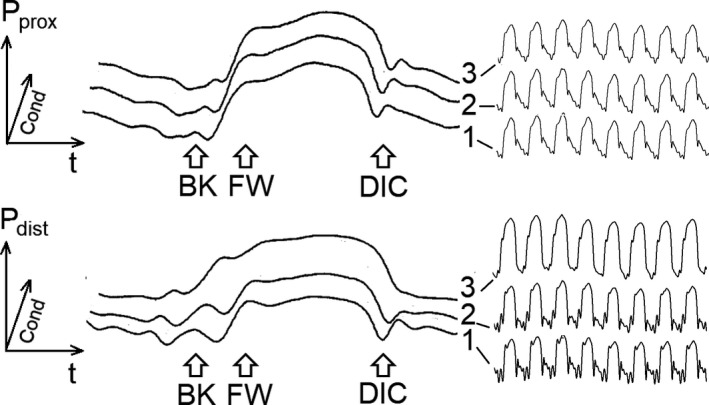
Impact of flow limitation on the amplitudes of the backward (BK) wave and of the dicrotic notch (DIC) in the proximal (*P*_prox_) and distal pressure (*P*_dist_) signals. Conditions 1, 2, and 3 are for baseline, flow limitation I (mild), and flow limitation II (moderate), respectively, at fixed hemodynamic conditions (heart rate = 110 bpm and aortic mean arterial pressure = 80 mmHg). All drawings show typical real traces: detail of a pressure wavefrom during one cardiac cycle at the left side and condensed concecutive pressure waveforms during a respiratory cycle at the right side

The validity of flow limitation was confirmed by FFR values: the median [IQR] FFR was 0.83 [0.75–0.89] for flow limitation I, and 0.73 [0.63–0.80] for flow limitation II. The FW method did not detect the forward wave at the distal side in the flow limitation condition, due to the higher amplitude of the backward wave during flow limitation (Figure 4), and CoPWV was therefore not calculated. Indeed, during stenosis the tangent to the forward wave was performed on a shorter segment which was in addition tilted by increased amplitude of the backward wave. Using the tangent intersection method induced an error in the determination of the onset of the forward wave. Similarly, the DIC method did no detect the dicrotic notch in the flow limitation condition, precluding the calculation of CoPWV. No significant difference was observed between flow limitation (I or II) and pre‐inflation period when CoPWV was assessed by the BK method. When measured by the TM method, the median CoPWV was significantly lower during flow limitation II than pre‐inflation (Table 4).

**TABLE 4:** Impact of flow limitation and stenting on CoPWV assessed by the four methods

	Median [IQR] CoPWV (m/s)				
	Pre‐inflation	Flow limitation I	Flow limitation II	Pre‐stenting	After stenting
Forward (FW)	4.9 [4.3–6.0]	–	–	8.9 [4.8–14.3]	3.6 [3.1–10.1]
Backward (BK)	4.1 [3.0–6.2]	4.5 [3.4–5.8]	4.1 [2.4–8.2]	4.9 [2.7–7.4]	5.4 [3.3–9.0]^†^
Dicrotic notch (DIC)	6.4 [6.2–7.4]	–	–	6.2 [6.1–6.9]	6.0 [5.3–7.0]
Template matching (TM)	6.6 [5.7–7.4]	5.2 [4.5–6.6]	4.9 [4.2–5.4]*	6.0 [5.5–6.7]	5.8 [4.5–6.4]

Note

Data are presented as median [interquartile range]. CoPWV, coronary pulse wave velocity.

* *p* ≤ .05 versus Pre‐inflation.

^†^ *p* ≤ .05 versus Pre‐stenting.

### 3.4 Condition 4: increased stiffness

Implanted stents had a length of 28 or 32 mm and a diameter of 2.5 or 2.75 mm to fit the arterial lumen. Stent implantation did not impede the use of any CoPWV measurement method. When measured by the BK method, the median CoPWV was significantly higher after stenting (Table 4).

## 4 DISCUSSION

The present study demonstrates that algorithms used to measure aortic or carotid PWV (FW and DIC methods) may be used for CoPWV measurement in healthy coronary arteries when blood flow is normal, but that these are not adapted to measure CoPWV in vessels presenting a reduction in blood flow. In the case of reduced blood flow, the BK and TM methods should be preferred since they allow the calculation of CoPWV in various hemodynamic conditions, that is, in normal conditions as well as in the presence of a reduced blood flow. It is of note, however, that BK and TM measure wave propagation at different states of the coronary artery (relaxed, i.e., less stiffer, vs. elongated, i.e., stiffer) and that although both provide interpretable results, these are not correlated. Therefore further work is required to determine which of these is the most informative, or if both are necessary for a complete characterization of coronary stiffness.

PWV in a uniform arterial segment is dependent on its stiffness but not on the direction of wave motion. Accordingly, CoPWV could be assessed by the propagation of the forward and backward waves, and both methods gave highly correlated values. The coronary backward wave originates from the contraction of the myocardial apex during isovolumic contraction (van Houwelingen et al., 2012). To the best of our knowledge, a backward wave has never been clearly identified on the intra‐coronary pressure itself except by using wave intensity analysis (Davies et al., 2006; Sen et al., 2014). This could be due to the fact that the sampling rate used in previous studies (0.1–1 kHz) is insufficient to clearly observe the backward wave. A previous observation from our group (Harbaoui et al., 2017) was made possible by a 2 kHz sampling, and the higher rate used herein (5 kHz) allowed an even more precise characterization of the backward wave. Conversely, a pressure perturbation due to isovolumic contraction was identified in the aorta (AIC) during isovolumic contraction (van Houwelingen et al., 2012). Whether AIC can also be observed at the proximal coronary level and thus interfere with the detection of the backward wave has never been documented. We consider that this is very unlikely due to the different travelling time of the AIC and backward pressure waves. The limits of backward wave detectability should be extensively validated in humans, especially in case of asynchronous/pathological myocardial contractions that could impact the quality of this waveform.

Regarding coronary stiffness, if it increases, as it is the case after elongation, PWV will increase. This has been reported for the carotid artery, with significantly lower PWV in early systole compared to end systole (Mirault et al., 2015), and observed herein; there was a trend toward higher CoPWV measured in the elongated artery (decompression: DIC and TM) than when this was measured in a relaxed artery (compression phase: FW). Furthermore, these methods were poorly correlated with the compression FW method. Of note, decompression methods had the best performance for period‐to‐period variability, which is an important feature for any potential clinical application.

Another condition in which stiffness should vary in relation to artery elongation, is a change in hemodynamic conditions, particularly blood pressure increase; herein, CoPWV increased with increasing MAP and this trend was consistent for all methods tested, which is similar to that reported in the coronary artery in dogs (Arts, Kruger, Gerven, Lambregts, & Reneman, 1979) and in other arteries (Nichols, O'Rourke, & Charalombos, 2011; Vermeersch, Dynamics, & Society, 2010). However, the velocity–pressure relationship in the coronary artery, which is a muscular artery, seems to have a shape (concave power function with exponent < 1) (Arts et al., 1979) different from the shape of the velocity‐pressure relationship observed in the aorta which is an elastic artery (convex power function with exponent > 1) (Nichols et al., 2011; Vermeersch et al., 2010). Indeed, for a 10 mmHg aortic MAP increase, CoPWV increased by 0.95 m/s from 60 to 80 mmHg and by 0.64 m/s from 80 to 100 mmHg with FW, by 0.52 m/s from 80 to 100 mmHg with BK, by 1.17 m/s from 60 to 80 mmHg and by 0.44 m/s from 80 to 100 mmHg with DIC, and by 1.17 m/s from 60 to 80 mmHg and by 0.55 m/s from 80 to 100 mmHg with TM method. Regardless of its shape, the fact CoPWV increased with increasing MAP has two implications: first, it strongly supports that CoPWV measurements are indices of stiffness, and secondly, that the variation of MAP required to produce meaningful changes of CoPWV markedly surpass those routinely observed during coronary catheterization. Thus, this pressure effect should not represent an issue for the clinical application of CoPWV. Conversely, the effect of HR was non‐significant, which is, again, consistent with previous reports that found that increasing HR was associated with a lower PWV change than increasing blood pressure (Albaladejo et al., 2001; Lantelme, Mestre, Lievre, Gressard, & Milon, 2002). From a clinical perspective, this is important since HR variations are frequent, but will not adversely affect the validity of the CoPWV value.

In the condition of flow limitation induced by balloon inflation, the backward wave became particularly noticeable at the distal coronary side; the resulting overlap between the backward wave and the forward wave prevents using the classical foot‐to‐foot method applied to the forward wave (*i.e.,* the FW method). The presence of the backward wave has previously been observed in human stenosed coronaries (called “premature pressure increase”) (Harbaoui et al., 2017), validating in a reverse manner the observation made in pigs in case of flow limitation: the amplitude of the backward wave decreased after flow restauration by angioplasty in humans (unpublished data). Due to a filtering effect, increasing flow limitation severity also smoothed the dicrotic notch, precluding the use of the DIC method to measure CoPWV in case of flow limitation. Only the BK and TM methods were able to provide data and only the TM method found a lowering of CoPWV with increasing flow limitation. Applying the Bramwell‐Hill equations (Nichols et al., 2011) to an annular lumen can explain this lowering: the subtraction of the constant balloon's cross‐sectional area from that of the artery's cross‐sectional area leads to a lower denominator but unchanged numerator as the area variation is dependent only on the artery diameter which is unchanged by balloon inflation. As the balloon induced a reduction of the lumen's cross‐sectional area by a median [IQR] of 31 [25–38]% (flow limitation I) and 38 [36–42]% (flow limitation II), CoPWV should decrease by a median [IQR] of 17 [14–21]% (flow limitation I) and 21 [20–24]% (flow limitation II) according to the Bramwell‐Hill equation. Moreover the lowering of the distal intra‐coronary pressure due to simulation of flow limitation should have a slight lowering effect according to the velocity–pressure relationship: lowering by a median [IQR] of 2 [0–11]% (flow limitation I) and 9 [6–15]% (flow limitation II). Thus, the overall predicted theoretical effect of a flow limitation should be a decrease by an approximate median [IQR] of 17 [14–34]% (flow limitation I) and 35 [22–41]% (flow limitation II), which is in line with obtained results: 18 [13–25]% (flow limitation I) and 29 [15–31]% (flow limitation II). This is discordant with what is expected to be observed in clinical practice for a true stenosis as the CoPWV should increase according to the Moens–Korteweg relationship (CoPWV is inversely proportional to the square root of the artery's diameter).

Importantly, the presence of two guidewires in the coronary should not have impacted the coronary flow: the sum of the cross‐section areas of the 2 guidewires of 0.014’’ = 0.35 mm is 2 * π * 0.1225/4 = 2 * 0.096 = 0.19 mm^2^ which is much lower than the cross section of the coronary artery measured by IVUS (range 6 – 13.5 mm^2^).

Stenting was used to increase the stiffness of the coronary artery, and only the BK method was able to detect a subtle increase of CoPWV, while the TM and other methods were unable to do so. Since the distal guidewire was jailed by the stent, pressure surge may have further reppeled the sensor in contact with the artery wall. This could have affected the upper part of the pressure wave and thus the related methods (TM and DIC methods), but it would have less likely impacted the lower part, explaining that the BK method was the only one providing the expected results. Nevertheless, the TM and DIC methods could have been equally affected during flow limitation by the smoothing of the pressure wave during the decompression fall (Figure 4) inducing a nonphysiological wave tilt. This smoothing was not observed on the backward wave which was, conversely, easier to detect due to its higher amplitude.

### 4.1 Limits

The porcine model of coronary circulation is widely accepted as representative of human coronary circulation. Of note, the various methods tested led to CoPWV values around 4–9 m/s, which are very close to those reported in dogs, especially for higher pressures (Arts et al., 1979). Indeed, applying a previously reported formula (CoPWV = 1.44 *P* ^0.69^, with *P* in kPa) obtained using a method similar to the FW method (Arts et al., 1979), the three levels of intra‐coronary pressure obtained in our experiments, yielded velocity values of 5.5 m/s, 6.5 m/s, and 7.6 m/s, values very similar to those obtained using the FW method (4.1 m/s, 6.0 m/s, and 7.3 m/s). Regarding the CoPWV obtained in humans, the DIC method yielded higher values (mean ± *SD*: 15.9 ± 1.8 m/s with) than those obtained herein in pigs (median [IQR] :5.8 [4.6–6.9] m/s) (Rolandi et al., 2014), which would be expected as the patients were elderly (68 ± 10) and the investigated arteries were not angiographically normal, both factors leading to an increase in CoPWV,

Implications concerning the use of pharmacologic agents on coronary stifness should be discussed. Norepinephrine has a known beta‐adrenergic effect at moderate/high doses, as observed in the present study, as well as a controversial possible vasoconstrictor effect on coronary arteries. Isosorbide dinitrate is a NO‐releasing vasodilator which also acts at the coronary level. Isoflurane also has a possible vasodilator coronary effect. It is thus highly possible that the mentioned pharmacological substances have a confounding effect on coronary stiffness, independently of the arterial pressure, but no other options were available to induce pressure variations.

The major limit of this model is, however, the fact that coronary arteries were non‐diseased, contrary to the potential clinical application envisioned for CoPWV. Healthy pigs were used in order to study the independent effects of hemodynamic variations, flow limitation, and increased stiffness on CoPWV. These experiments should be reproduced in a porcine model of advanced coronary atherosclerosis. Furthermore, we used a model of coronary flow limitation that is published (Young et al., 2013), but that may not compare to true vessel wall thickening. Another potential limitation is that stenting as an experimental model of coronary stiffening may not lead to a sufficient change of stiffness; stents were rather short and expanded within a normal artery. Furthermore, the distal wire was jailed by the stent, potentially disturbing pressure signal at high pressure. This may explain why only one method was able to reproduce the results obtained in humans in whom stenosis relief and stenting were associated with an increase of CoPWV (Harbaoui et al., 2017).

## 5 CONCLUSION

Using a comprehensive analysis of wave propagation, the present study found that CoPWV can be measured by various methods using different characteristic points. Among those tested, two were found to be of potential intersest for further studies in humans.

## CONFLICT OF INTEREST

AC, BH, CC, PYC, and PL are shareholders of I‐COR Technologies exploiting two patents concerning the computation of the coPWV. The remaining authors report no relationships that could be construed as a conflict of interest.

## AUTHOR CONTRIBUTIONS

AC, BH, and PL conceived and planned the experiments. AC, BH, CC, JMB, CP, PYC, and PL carried out the experiments. AC performed the data analysis. AC and PL performed the statistical analysis. AC, BH, CC, PYC, and PL contributed to the interpretation of the results. AC took the lead in writing the manuscript. AC and PL wrote the manuscript in consultation with BH and PYC. All authors provided critical feedback and helped shape the research, analysis, and manuscript.

## Appendix 1

### METHODS ‐ DETAILED DESCRIPTION

#### Anesthesia and ventilation

Animals were sedated with an intramuscular administration of a 1:1 mixture of tiletamine and zolazepam (Zoletil™ 100, 100 mg/ml, Virbac, Carros, France), 3.0 mg/kg and morphine 0.1 mg/kg. Induction was carried out with propofol (Propovet, 10 mg/ml, Axience, Pantin, France) 4.0 mg/kg intravenously. After induction of anesthesia, animals were orotracheally intubated and placed under mechanical ventilation with the controlled tidal volume set at 8.0 ml/kg and a respiratory rate of 15 cycles/min. Heart rate was monitored from ECG (screw electrodes inserted in the skin) using a patient monitor (MX700 IntelliVue, Philips, Boeblingen, Germany). Maintenance of anesthesia was ensured by volatile anesthesia with sevoflurane (SevoFlo, Axience, Pantin, France). Intravenous Ringer lactate was administered during the experiment at a basal rate of 10 ml kg^−1^ h^−1^.

#### Vascular approach, temporary pacemaker insertion, and coronary angiogram

The skin at the operation site was cleansed with antiseptic solution and the neck covered with sterile drapes to keep the incision area as clean as possible. A right and a left lateral cervicotomy were performed to isolate the jugular vein and the carotid artery on the right side, and the contralateral jugular vein on the left side. A central venous catheter (6 French) was inserted in the left jugular vein for fluid and drug administration. An additional 6 French desilet was inserted into the right jugular vein for the external atrial pacing at the three selected heart rates.

An arterial catheter (7 French, Aeris™, Abbott, Saint‐Paul, MN, US) was inserted into the carotid artery allowing to introduce the coronary angiography probe 7 French (JR4 or JL3,5) into the LAD. 10 000 Units of Unfractionated Heparin (Panpharma, Luitré, France) and 250 to 500 mg of Aspirin (Sanofi‐Aventis, Gentilly, France) were administrated intravenously. Contrast injection (iobitridol, Guerbet, Roissy, France) was used for testing of the appropriate position. Isosorbide dinitrate (1 mg) (Sanofi‐Aventis, Gentilly, France) was administered intra‐coronarily before the start of experiments in order to avoid coronary spasm that may occur during a coronary angiogram and to counteract the effects of the other drugs used during experiments. The left coronary artery was chosen because of its similarity to the human coronary artery.

A catheter‐based invasive blood pressure measurement tool was inserted until the proximal side of the angiography probe and then connected to the same Philips patient monitor used for the ECG monitoring. To avoid catheter clotting, a heparinized sodium chloride flush was done every minute and before each recording. Two concomitant signals of intra‐coronary pressure were obtained using two identical FFR guidewires (Aeris™, Abbott) each connected to a distinct FFR integrated system of same manufacturer's reference (Quantien™, Abbott).

#### Data filtering during acquisition

During acquisition, the acquired waveforms were digitally filtered: ECG‐baseline and power‐line and low‐pass filter (Finite Impulse Response – FIR filter, Blackman window, 60 Hz, 201 coefficients). *P*_prox_ and *P*_dist_: low‐pass filter (FIR filter, Blackman window, 30 Hz, 701 coefficients).

#### Data pre‐processing

The pre‐processing of acquired data by the RECAN software (Alpha‐2, Lyon, France) are described below. Beat‐by‐beat HR and systolic and diastolic *P*_dist_ were automatically calculated and manually edited afterwards in order to exclude extrasystoles or periods with disturbed signals. The graphical user interface of RECAN allowed visual selection of the most stable 1‐min analysis periods and of the nadir of *P*_dist_ after adenosine administration. First derivatives of *P*_prox_ and *P*_dist_ were computed for each sample using a three‐point central difference algorithm. A positive threshold automatically adjusted on the maximum of *P*_prox_ and *P*_dist_ derivatives during a cardiac cycle allowed detection of the onset of the initial pressure rise in *P*_prox_ and *P*_dist_ signals. A negative threshold automatically adjusted on the minimum of *P*_prox_ and *P*_dist_ derivatives during a cardiac cycle allowed detection of the onset of the pressure fall after the end of the cardiac contraction in *P*_prox_ and *P*_dist_ signals.

#### Computing of the pressure wave transit time

The four methods for the computation of the pressure wave transit time between the two pressure sensors are detailed below. *Forward method (FW)*: The forward wave was considered to be the initial pressure rise detected in *P*_prox_ and *P*_dist_ signals (Figure 2) using their derivatives during preprocessing. The tangent intersection method was adapted to the forward wave as follows. A linear regression was performed on a segment of 30 ms centered on the maximum of the pressure derivative corresponding to the forward wave. As long as the regression coefficient was lower than 0.999, the regression was repeated on the segment shifted towards posterior times by a 2 ms increment until the segment reached the maximum of the pressure or the regression coefficient was higher than 0.999. If the maximum regression coefficient during all the iteration was lower than 0.750, the corresponding cardiac cycle was discarded from further analysis, otherwise, the regression with the maximum regression coefficient was memorized. The natural logarithm of the pressure fall during diastole of the precedent cardiac cycle was calculated. A linear regression was calculated on a segment of the pressure logarithm starting after the dicrotic notch of the precedent cardiac cycle and ending before the onset of the pressure rise of the current cardiac cycle. If the regression coefficient of this linear regression was high enough, the diastolic pressure was given by the extrapolation of this regression at the moment of the pressure minimum before the onset of the forward wave, otherwise, the diastolic pressure was the pressure minimum before the onset of the forward wave. The intersection point between the tangent to the forward pressure rise and the diastolic pressure was considered to be the final value of the foot of the forward wave. The interval of time between the foot of the forward wave of *P*_prox_ and the foot of the forward wave of *P*_dist_ was considered to be the transit time of the compression forward wave.

*Backward method (BK)*: The backward wave was detected as follows. The minimum of the *P*_prox_ and *P*_dist_ derivatives was detected just before the onset of the initial pressure rise in *P*_prox_ and *P*_dist_ signals detected during preprocessing. The maximum of the *P*_prox_ and *P*_dist_ derivatives corresponding to the backward pressure rise was detected just before the minimum *P*_prox_ and *P*_dist_ derivatives within 70 ms. If this maximum of pressure derivative was not positive or not distinct from the following minimum of the pressure derivative, the final/definite maximum of the pressure derivative corresponding to the backward wave was those detected during preprocessing and corresponding to the onset of the initial pressure rise. The tangent intersection method was adapted to the backward wave for the *P*_prox_ and *P_dist_* signals as follows (Figure 2). A linear regression was performed on a segment of 25 ms centered on the maximum of the pressure derivative corresponding to the backward wave. While the regression coefficient was lower than 0.999, the regression was repeated on the segment shifted towards anterior times by a 2 ms increment until the segment reached the minimum of the pressure or the regression coefficient was higher than 0.999. If the maximum regression coefficient during all the iteration was lower than 0.750, the corresponding cardiac cycle was discarded from further analysis, otherwise, the regression with the maximum regression coefficient was memorized. The natural logarithm of the pressure fall during diastole of the precedent cardiac cycle was calculated. A linear regression was calculated on a segment of the pressure logarithm starting after the dicrotic notch of the precedent cardiac cycle and ending before the onset of the pressure rise of the current cardiac cycle. If the regression coefficient of this linear regression was high enough, the diastolic pressure was given by the extrapolation of this regression at the moment of the pressure minimum before the onset of the backward wave, otherwise, the diastolic pressure was the pressure minimum before the onset of the backward wave. The intersection point between the tangent to the backward pressure rise and the diastolic pressure was considered to be the final value of the foot of the backward wave. The delay between the foot of the backward wave of *P_dist_* and the foot of the backward wave of *P*_prox_ was considered to be the transit time of the compression backward wave.

*Dicrotic notch method (DIC)*: The dicrotic notch was detected as follows. The nadir of the *P_dist_* and *P*_prox_ derivatives was detected immediately after the onset of the pressure fall detected during preprocessing. The second derivative of each pressure signal was calculated on the 60 ms segment between the nadir and the following maximum of the pressure first derivative using a three‐point‐stencil central difference algorithm applied to the averaged (10‐points moving average) first derivative. The maximum of the second derivative on this segment was considered to be the dicrotic notch (Figure 2). The delay between the dicrotic notch of *P*_prox_ and *P_dist_* was considered to be the transit time of the decompression pressure wave. *Template matching (TM)*: The template matching method was applied as follows. The amplitudes of the nadir of *P*_prox_ and *P_dist_* derivatives were calculated. A segment was delimitated on *P*_prox_ and *P_dist_* fall, starting with the point where pressure derivative was 30% of the nadir's amplitude and ending at the point corresponding to the dicrotic notch. The two segments delimitated on *P*_prox_ and *P_dist_*, were rescaled with a coefficient equal to the ratio of the derivative nadir's amplitudes and then shifted sample by sample in order to obtain the best template matching. The shift between the two segments corresponding to the best template matching was considered to be the transit time of the decompression pressure wave (Figure 2).

#### Filtering of abnormal transit times

Transit times outside the interval [4–40] ms were considered abnormal and were excluded. Then, the mean of all the remaining transit delays was calculated during a 1‐min period and only the values in the interval of ±33% of the 1‐min mean were kept. Finally, the distance between the sensors was divided by the filtered values of the transit delay, resulting in a time series of instantaneous CoPWV for each cardiac cycle.
